# Spatial Heterogeneity in Myelin Sheathing Impacts Signaling Reliability and Susceptibility to Injury

**DOI:** 10.1523/ENEURO.0402-24.2025

**Published:** 2025-02-14

**Authors:** Afroditi Talidou, Jérémie Lefebvre

**Affiliations:** ^1^Department of Cell Biology and Anatomy, University of Calgary, Calgary, Alberta T2N 4N1, Canada; ^2^Department of Biology, University of Ottawa, Ottawa, Ontario K1N 6N5, Canada; ^3^Krembil Brain Institute, University Health Network, Toronto, Ontario M5T 0S8, Canada; ^4^Department of Mathematics, University of Toronto, Toronto, Ontario M5S 2E4, Canada

**Keywords:** conduction delays, conduction failure, demyelination, motifs, myelin, myelin sheath

## Abstract

Axons in the mammalian brain show significant diversity in myelination motifs, displaying spatial heterogeneity in sheathing along individual axons and across brain regions. However, its impact on neural signaling and susceptibility to injury remains poorly understood. To address this, we leveraged cable theory and developed model axons replicating the myelin sheath distributions observed experimentally in different regions of the mouse central nervous system. We examined how the spatial arrangement of myelin affects propagation and predisposition to conduction failure in axons with cortical versus callosal myelination motifs. Our results indicate that regional differences in myelination significantly influence conduction timing and signaling reliability. Sensitivity of action potential propagation to the specific positioning, lengths, and ordering of myelinated and exposed segments reveals non-linear and path-dependent conduction. Furthermore, myelination motifs impact signaling vulnerability to demyelination, with callosal motifs being particularly sensitive to myelin changes. These findings highlight the crucial role of myelinating glia in brain function and disease.

## Significance Statement

This study highlights the importance of spatial heterogeneity of myelin sheathing in shaping nerve axonal conduction. Using model axons that faithfully replicate myelin distributions observed in the mouse central nervous system, our results revealed the impact of myelin patterns on the timing and reliability of neural signaling. Contrary to the conventional view in which uniform and equally spaced myelin segments imply linear conduction, our findings show that axonal conduction is a path-dependent, nonlinear process influenced by the specific distribution of myelinated and unmyelinated segments. Membrane potential propagation along model axons in the corpus callosum was found to be more vulnerable to myelin changes compared to those in the cortex, especially post-myelin injury, emphasizing the role of sheath locations in both health and disease.

## Introduction

Myelinated axons play a pivotal role in supporting and facilitating the faithful conduction of action potentials (APs). Myelin-forming oligodendrocytes (OLs) engage in the synthesis of cellular membranes enwrapping axons through a multilamellar spiral configuration (Bradl and Lassmann, 2010; Simons and Nave, 2015), serving as an effective insulator for the axons and enabling saltatory conduction. Individual OLs have the capacity to generate multiple myelin sheaths, with each sheath potentially varying in length and enwrapping different axons (Chong et al., 2012; Tomassy et al., 2014). In contrast to the conventional depiction of axons, where myelin sheaths are uniformly distributed along their length, numerous investigations have shown that axonal myelin sheathing is, in fact, highly heterogeneous. Even when controlling for variations in length and overall myelin coverage (that is, the percentage of the axon covered by myelin, referred thereafter as PMC), internodes’ number, thickness, length and spatial arrangement do vary significantly between and along given axons (Sturrock, 1980; Bakiri et al., 2011; Tomassy et al., 2014; Ford et al., 2015; Micheva et al., 2016; Auer et al., 2018; Hill et al., 2018; Stedehouder et al., 2019; Benamer et al., 2020; Call and Bergles, 2021). Such diversity extends within and across brain regions (Glasser and Essen, 2011; Thornton et al., 2024). One of the most salient differences can be observed when comparing myelination motifs found in the white matter and cortex (Geurts and Barkhof, 2008; Glasser and Essen, 2011; Tomassy et al., 2014; Calabrese et al., 2015; Prins et al., 2015; Micheva et al., 2016; Timmler and Simons, 2019; Orthmann-Murphy et al., 2020; Call and Bergles, 2021; Corrigan et al., 2021; Lie et al., 2022; Thornton et al., 2024). Indeed, as axons traverse the corpus callosum, internode lengths exhibit greater variability, whereas, in the cerebral cortex, they tend to be more uniform (Chong et al., 2012). Myelination in the cortex and the corpus callosum follow independent developmental trajectories (Corrigan et al., 2021), further exhibiting distinct growth and regenerative responses following injury (Orthmann-Murphy et al., 2020; Thornton et al., 2024). Variability in myelin sheathing motifs also extends to brain regions and cell types (Micheva et al., 2016). Such striking differences are of prime functional significance, suggesting that precise sheath placement is important for cortical function (Orthmann-Murphy et al., 2020), reflecting regional differences in myelination strategies to support neural signaling (Chong et al., 2012; Tomassy et al., 2014; Thornton et al., 2024). Indeed, the spatial heterogeneity resulting from the particular positions and lengths of myelin sheaths, the molecular constitution of nodes, along with variations in the distribution and density of ion and/or leak channels, collectively influence axonal conduction and the reliable propagation of APs (Osso and Hughes, 2024). Such microstructural changes notably tune axonal conduction delays – the temporal interval required for an AP originating at the axon hillock to reach post-synaptic targets – which play an important role in neural circuits operation (Fields, 2008; Talidou et al., 2022) and to maintain brain function (Nagy et al., 2004; Bengtsson et al., 2005; Mabbott et al., 2006; Pujol et al., 2006; McKenzie et al., 2014; Pan et al., 2020; Steadman et al., 2020; Xin and Chan, 2020).

A crucial question that remains unanswered is the impact of such heterogeneity on the conduction of APs. Does spatial variation in myelin sheathing pattern affect conduction delays? If so, how much? Are these patterns equally resilient to variations in myelination, or are certain motifs more fragile and prone to conduction failure with changes in myelin coverage? Understanding the implications of regional myelination motifs diversity on axonal conduction would represent an important advancement in our understanding of the tactics employed by OLs to optimize neural trafficking, and in identifying which axons or part of these axons are more vulnerable to injury. An extensive corpus of scientific literature has developed mathematical models to investigate the propagation of APs along myelinated axons (Fitzhugh, 1962; Goldman and Albus, 1968; Grindrod and Sleeman, 1985; Basser, 1993; Nygren and Halter, 1999; McIntyre et al., 2002; Gow and Devaux, 2008; Babbs and Shi, 2013; Ashida and Nogueira, 2018; Scurfield and Latimer, 2018; Naud and Longtin, 2019; Schmidt and Knösche, 2019). Prevailing models oftentimes presuppose uniform, homogeneous myelin sheathing interspersed with exceedingly brief nodes of Ranvier. However, this convenient approximation severely limits the characterization of the effect of spatial heterogeneity in myelin sheathing along axons (Chong et al., 2012; Tomassy et al., 2014).

To circumvent this limitation, we developed model axons endowed with myelin sheath patterns closely matching those measured from the cerebral cortex and corpus callosum of mice (Chong et al., 2012). In vivo studies conducted in these regions (and others) by Chong et al. (2012) revealed considerable variability in the number, length and arrangement of myelin sheaths formed by individual OLs, representing ideal starting points to ground our explorations. We hence used these data to constrain our model axons, and compared how these distinct myelination motifs shape AP conduction reliability, as well as vulnerability to failure during demyelination. Our approach integrates variability in the lengths and number of myelin sheaths and exposed segments while keeping other parameters (e.g., axonal length, radius, g-ratio, biophysical parameters, etc) constant. This was done to ensure that any observed differences in axonal conduction can be attributed exclusively to myelin placement along the axons. We used and adapted cable theory, amenable to the representation of AP propagation across axons composed of myelin sheaths and exposed segments of arbitrary (and different) lengths. To face the challenging computational demands of analyzing such physiologically realistic axons, we leveraged a recent modeling framework (Grindrod and Sleeman, 1985; Ashida and Nogueira, 2018; Naud and Longtin, 2019; Schmidt and Knösche, 2019), in which a phenomenological model of membrane excitability is used to reproduce the rapid depolarization (spike) of the membrane potential along the exposed segments of the axon (Ashida and Nogueira, 2018), combined with diffusion accounting for the fast transmission of APs within myelin sheaths. This framework, shown to faithfully simulate propagation along myelinated axons (Ashida and Nogueira, 2018; Schmidt and Knösche, 2019), allowed us to quantify how spatial heterogeneity in myelin sheathing influences AP conduction, as well as the occurrence of conduction failures and susceptibility to conduction block.

## Methods and Materials

### Constructing axons with heterogeneous myelination motifs

We consider a cable of fixed length *L*, representing a bare axon. The cumulative length of the cable covered by myelin (*L*_*my*_) is determined by the formula *L*_*my*_ = *PMC* · *L*, where *PMC* represents the percentage of myelin coverage. The remaining part of the axon constitutes the exposed length (*L*_*exp*_), expressed as *L*_*exp*_ = *L* − *L*_*my*_. To construct a myelinated axon, we generate myelin sheaths, each of length $l_{my}^{i}$ (*i* = 1, …, *N*_*my*_), and exposed segments $l_{exp}^{i}$ (*i* = 1, …, *N*_*exp*_), which are assembled in an alternating fashion. *N*_*my*_ and *N*_*exp*_ represent the total number of myelinated and exposed segments, respectively. Geometrically, the lengths *L*_*my*_ and *L*_*exp*_ correspond to the sum of individual myelinated or exposed segment lengths, as follows:

$$L_{my}=\sum_{i=1}^{N_{my}}l_{my}^{i},$$

$$L_{exp}=\sum_{i=1}^{N_{exp}}l_{exp}^{i}.$$

The distribution of myelin sheaths with variable lengths is carried out along the cable, as detailed in the subsequent analyses.

#### Cortical myelination motifs

To create an axon with cortical-like myelination we generate a set of Poisson-distributed numbers that sum to *L*_*my*_. Each element of the set corresponds to a myelin sheath with the value of the element denoting the length of the sheath $l_{my}^{i}$. To generate this set, we first generate *L*_*my*_ points uniformly on the unit interval. In each sub-interval of length 1/*N*_*my*_, where *N*_*my*_ is the number of sheaths, there are on average *L*_*my*_/*N*_*my*_ events. Hence, there will be *N*_*my*_ events in total and the Poisson rate parameter becomes *λ* = *L*_*my*_/*N*_*my*_. We subsequently check whether the following constraints are satisfied: we require that the mean value of the sheath lengths is between 30 μm and 100 μm according to Chong et al. (2012) and Call and Bergles (2021), and the minimal length is no smaller than 1 μm (Call and Bergles, 2021). The lengths of exposed segments were generated in the same manner, with the additional constraint that the minimum segment length is 1 μm, a value commonly associated with a node of Ranvier (Arancibia-Cárcamo et al., 2017).

#### Callosal myelination motifs

To create an axon with callosal-like myelination we adopt a similar strategy, but use a different distribution compared to the one used above. We generate a set of Gamma-distributed numbers (*N*_*my*_ in total) that sum to *L*_*my*_. The rationale underlying the selection of the Gamma distribution stems from the irregular sheath lengths generated per oligodendrocyte within the corpus callosum observed experimentally (Chong et al., 2012). Each element of a set following the Gamma distribution, Γ(*k*, *θ*), corresponds to a myelin sheath with the value of the element denoting the length of the sheath ( $l_{my}^{i},i=1,\ldots ,N_{my}$). The shape parameter *k* and the scale parameter *θ* vary for each axon and are sampled from the uniform distribution. We have set two constraints for axons with callosal motifs: (i) the mean value of the sheath lengths is between 20 and 160 μm, and (ii) the minimal sheath length is greater or equal to 1 μm. We generate the exposed segment lengths in the same manner as with the myelin sheaths. The constraint is the same as for the cortical axons, namely that the minimal length of an exposed segment is 1 μm.

### Modeling AP propagation along heterogeneously myelinated axons

To address the computational challenges of analyzing physiologically realistic axons, we combined elements from recent studies simulating AP conduction along myelinated axons (Grindrod and Sleeman, 1985; Ashida and Nogueira, 2018; Naud and Longtin, 2019; Schmidt and Knösche, 2019). These approaches, use simplified phenomenological models of rapid, standardized depolarization (spike) of the membrane potential along exposed segments, combined with the cable equation which describes the diffusion of AP along both exposed segments and myelin sheaths. Such models have repeatedly been shown to be in agreement with more detailed biophysical models relying on the Hodgkin-Huxley formalism (e.g., Arancibia-Cárcamo et al., 2017).

The membrane potential is determined by the properties and particular structure of myelinated and exposed segments along an axon. Due to high resistance along myelin sheaths, the membrane potential travels passively there and regenerates over exposed segments characterized by higher density of ion channels. Taking these properties into consideration, we built a model that governs the dynamics of the membrane potential along different segments of the axon. The model is defined by the following system of partial differential equations and conditions:

$$C_{c}\frac{\partial u_{i}}{\partial t}(x,t)=\frac{a}{2r_{int}}\frac{\partial^{2}u_{i}}{\partial x^{2}}(x,t)+I(u_{i}(x,t)),$$

$$C_{my}\frac{\partial u_{i+1}}{\partial t}(x,t)=\frac{1}{R_{c}}\frac{\partial^{2}u_{i+1}}{\partial x^{2}}(x,t),$$

$$u_{i}(p_{i},t)=u_{i+1}(p_{i+1},t),$$

$$\kappa_{i}\frac{\partial u_{i}}{\partial x}(p_{i},t)=\kappa_{i+1}\frac{\partial u_{i+1}}{\partial x}(p_{i+1},t),$$

$$\frac{\partial u}{\partial x}(0,t)=0,\;\frac{\partial u}{\partial x}(L,t)=0,$$

$$u(x,0)=E_{leak},$$

for *i* = 1, …, *N*, where *u*_*i*_(*x*, *t*), 0 < *x* < *L*, is the membrane potential within segment *i* (either myelinated or exposed/unmyelinated) at time *t* > 0 and for a total of *N* = *N*_*my*_ + *N*_*exp*_ segments.

The solution *u*_*i*_ of each differential equation involved in Equations 3–4 is the membrane potential within either an exposed segment/node of Ranvier (Eq. 3) or a myelin sheath (Eq. 4). We imposed matching conditions at the paranodes (see Eqs. 5–6), namely at the regions where the terminal myelin loops form septate-like junctions with the axolemma. Equation 5 indicates that the membrane potential *u* is continuous across a paranodal junction while Equation 6 assumes continuity in flux, that is, the longitudinal currents should match at the junction. Following Rall (1969), we assume zero Neumann boundary conditions at either end (Eqs. 7). For time *t* = 0 we set an initial condition equal to the leak reversal potential *E*_*leak*_ (Eq. 8). We analyze the resulting governing equations and define all the parameters below.

#### Exposed segments/nodes of Ranvier

Equation 3 models the membrane potential *u* along the unmyelinated parts of the axon. This model has been introduced in Table 5 in Ashida and Nogueira (2018) as a simplified version of the Hodgkin and Huxley (1952) and Wang–Buzaki models (Wang and Buzsáki, 1996). It accounts for spike generation and conduction, and has been inspired by the exponential integrate-and-fire model, which simulates the rapid growth of the membrane potential. In Equation 3, the membrane capacitance of the cable is denoted by *C*_*c*_, *a* is the radius of the cable and *r*_*int*_ is the intracellular resistivity. Three currents are lumped together into I(u): the leak current (*I*_*leak*_), the depolarizing current (*I*_*dep*_), and the repolarizing current (*I*_*rep*_). The leak current is given by

$$I_{leak}(u(x,t))=g_{leak}(E_{leak}-u(x,t)),$$

where *g*_*leak*_ is the leak conductance and *E*_*leak*_ the leak reversal potential. Both parameters were assumed to remain constant along the axon. The depolarizing current emulates the spike initiation and is defined as follows:

$$I_{dep}(u(x,t))=g_{leak}\frac{K_{th}A_{th}}{1+A_{th}e^{-U_{th}}},$$

where $U_{th}=\frac{u(x,t)-u_{th}}{K_{th}}$. The parameter *u*_*th*_ is the spike threshold, and *K*_*th*_, *A*_*th*_ are parameters needed to control the exponential growth of the spike. When the membrane potential *u* reaches the preset value *u*_*rep*_, a repolarizing current is triggered forcing the membrane potential downward to the resting state. The repolarizing current is defined as:

$$I_{rep}(u(x,t))=G_{rep}(t)g_{leak}(E_{leak}-u(x,t)),$$

with *G*_*rep*_(*t*) being the repolarizing conductance for a time *t* ≥ *T*_*rep*_ (the time of activation of the repolarizing current):

$$G_{rep}(t)=A_{rep}\frac{t-T_{rep}}{t_{rep}}e^{1-\frac{t-T_{rep}}{t_{rep}}}.$$

The parameters *A*_*rep*_ and *t*_*rep*_ are the amplitude and time constants of the repolarizing conductance, respectively.

To generate an AP, a brief external current *I*_*inj*_ is injected into the second exposed segment of the axon for a set duration *T*_*S*_:

$$I_{inj}(x,t)=\left\{ \begin{array}{cc} I_{0}, & x=x_{0},\;t\in T_{S}\\ 0, & \text{elsewhere}. \end{array}\right.$$

For a thorough discussion of the model used in Equation 3, and its advantages and disadvantages compared to the classical conduction models, we refer the reader to the original work (Ashida and Nogueira, 2018). The parameter values used here are also taken from Ashida and Nogueira (2018) and are summarized in Table 1.

**Table 1.** List of model variables and parameters

Symbol	Definition	Value
*L*	axonal length	10 mm
*L*_*my*_	myelinated axon length	variable
*L*_*exp*_	exposed axon length	variable
*l*_*my*_	length of a myelin sheath	variable
*N*_*my*_	number of myelin sheaths	variable
*l*_*exp*_	length of an exposed segment	variable
*N*_*exp*_	number of exposed segments	variable
*N*	total number of segments	variable
*λ*	Poisson parameter	variable
*k*	shape parameter of Gamma distribution	variable
*θ*	scale parameter of Gamma distribution	variable
*C*_*c*_	cable membrane capacitance	3.5 pF/mm^2^
*a*	cable radius	2 μm
*r*_*int*_	intracellular resistivity	100 MOhm·μm
*E*_*leak*_	leak reversal potential	−68.3 mV
*g*_*leak*_	leak conductance	1 mS/cm^2^
*K*_*th*_	slope factor	3.5 mV
*A*_*th*_	ceiling factor	520
*u*_*th*_	spike threshold	−60.2 mV
*u*_*rep*_	starting voltage of repolarizing current	10 mV
*t*_*rep*_	time constant of repolarizing conductance	1 ms
*A*_*rep*_	amplitude constant of the repolarizing conductance	90
*I*_0_	amplitude of the injected current	0.01 nA
ϵ	permittivity of myelin	1.4 · 10^−10^ F/m
*g*	*g*-ratio	0.6

#### Myelin sheaths

Turning to the parts of the axon covered by myelin, we assume that the myelin sheaths have infinite resistance. Consequently, in the absence of ionic currents, the membrane potential *u* along a myelin sheath satisfies the diffusion equation Equation 4 (cf. Dayan and Abbott, 2005). The capacitance of a myelin sheath *C*_*my*_ is defined as follows:

$$C_{my}=\frac{2\pi \epsilon }{\ln\;(1/g)},$$

where ϵ is the permittivity of the myelin and *g* is the *g*-ratio, that is, the ratio of the inner-to-outer diameter of the myelinated axon. The cable resistance is given by the intracellular resistivity *r*_*int*_ over the cross-sectional area $\pi a^{2}$, that is,

$$R_{c}=\frac{r_{int}}{\pi a^{2}}.$$

#### Paranodal junctions

As the myelin loops bind to the axonal membrane, they form paranodal axoglial junctions at both ends of every myelin sheath. While there is a rich selection of dynamics and functions at the paranodal junctions to maintain reliable saltatory conduction (Rosenbluth, 2009) we exclude those details from our model. We apply the matching conditions given in Equations 5–6 at each paranodal junction, here denoted by π. The diffusivities κ in Equation 6 are defined by the diffusion coefficients of Equations 3–4. Specifically, from Equation 3 we have $\kappa_{i}=\frac{1}{C_{c}}\frac{a}{2r_{int}}$ and from Equation 4 $\kappa_{i+1}=\frac{1}{C_{my}}\frac{1}{R_{c}}$.

### Axonal conduction delays, CVs and conduction failure

For each segment (either myelinated or exposed/unmyelinated) along an axon, we compute the difference between the time the membrane potential spikes at the beginning and end of the segment. This difference gives the conduction delay along individual segments referred thereafter as segmental delay. We denote these delays by $\tau_{my}^{i}$, *i* = 1, …, *N*_*my*_, and $\tau_{exp}^{i}$, *i* = 1, …, *N*_*exp*_, corresponding to the conduction times across individual myelinated and exposed segments, respectively. Then, the conduction delay *τ* along the entire axon is given by the sum of segmental delays:

$$\tau =\sum_{i=1}^{N_{my}}\tau_{my}^{\;i}+\sum_{i=1}^{N_{exp}}\tau_{exp}^{\;i},$$

where we recall that *N*_*my*_ and *N*_*exp*_ denote the total number of myelinated and exposed segments along a single axon, respectively. Note that since APs propagate rapidly along myelin sheaths due to diffusion, the overall conduction delay τ in Equation 16 predominantly reflects propagation time along exposed segments, that is, $\tau \approx \sum_{i=1}^{N_{exp}}\tau_{exp}^{i}$. To obtain the corresponding conduction velocity (CV) we divide the total axonal length *L*, which is also equal to the sum of myelin sheaths and exposed segment lengths, by the conduction delay τ:

$$CV=\frac{L}{\tau }.$$

To examine whether an AP successfully conducts or fails to transverse the entire axon, we check the AP peak value at the end of each segment (myelinated or not). If the AP peak value is below the threshold value $U_{th}$ (see Eq. 10), then we have a conduction failure.

### Simulating myelin injury

We model demyelination along individual axons by removing 5% of the number of myelin sheaths. We repeated this process six times (D1, …, D6). Demyelination was conducted by randomly selecting myelin sheaths that had not been removed in previous steps. Once a myelin sheath was removed from the axon in any demyelination step, it remained impaired throughout subsequent iterations. In the event of myelin sheath damage, the damaged sheath and the adjacent nodes become a newly exposed segment which is modelled using Equation 3 for excitable media. Additionally, to explore the implications of sodium channel density on axonal conduction during demyelination we vary the AP threshold value along the newly formed exposed segments. The AP threshold values range between −65 mV to −25 mV. The AP threshold along the unaffected exposed segments/nodes of Ranvier remains constant at the baseline value (−60.2 mV). The threshold value serves here as a proxy for the reduced excitability of the axon, reflecting a decrease in the density of sodium ion channels; an increase in channel density corresponds to a lower AP threshold.

We compute axonal conduction delays ( τ) by summing the conduction delays across individual myelinated ( $\tau_{my}^{i}$) and exposed ( $\tau_{exp}^{i}$) segments (including the newly formed exposed segments) as in Equation 16.

### Numerical simulations

We modelled an axon as a medium composed of multiple layers, each representing the myelinated and exposed segments of the axon. Each segment was discretized into *n* + 1 equally spaced compartments, and second-order finite differences were used in space. Due to the varying lengths of the segments, the spatial step size *dx* differed for each segment. For temporal integration, we used the implicit Euler method.

To mitigate boundary effects at the beginning and end of the axon, we extend the axon at both ends, using copies of a portion of the axon with the same spatial and biophysical properties. This approach allowed us to compute the conduction delays and velocities along fixed axonal lengths without boundary artifacts. The numerical schemes used for the approximation of the solution of Equations 3–8 were based on the methods proposed by Carr and Turner (2016) and Hickson et al. (2011), which address the change of dynamics at the interfaces, namely at the paranodal junctions. The numerical scheme was implemented in MATLAB code, which is available online at *https://github.com/atalidou/myelinPatterns*.

## Results

### Modeling axons with spatially heterogeneous myelin sheathing

**Figure 1.**
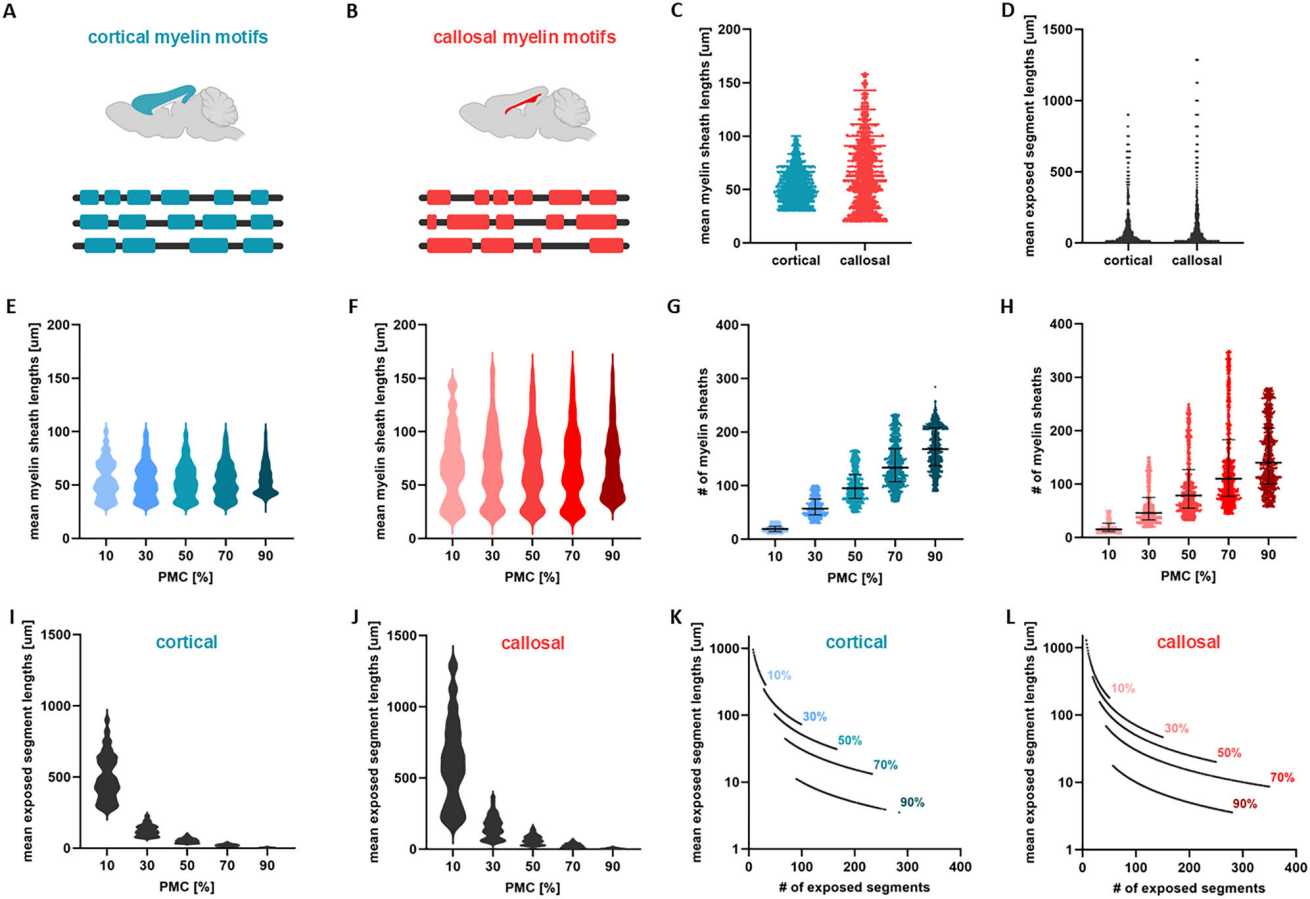
Modeling axons of heterogeneous myelination motifs in grey and white matter. ***A***,*** B***, Schematic illustration of myelination motifs along individual axons in two different brain regions: in the cerebral cortex (blue, panel *A*) and corpus callosum (red, panel *B*). Callosal motifs are more spatially heterogeneous compared to those found in the cortex. ***C***, Scatter plot of the mean values of myelin sheath lengths for axons featuring cortical and callosal myelin motifs for all cases of PMC examined. ***D***, Scatter plot of the mean values of exposed segment lengths. ***E–F***, Mean values of myelin sheath lengths, corresponding to cortical (panel *E*) and callosal (panel *F*) motifs, plotted separately for each PMC in the form of violin plots. ***G–H***, Scatter plots of the number of myelin sheaths as a function of PMC (cortical motifs - panel *G*, callosal motifs - panel *H*). The lines indicate the median with interquartile range of the plotted data. ***I–J***, Violin plots of the mean values of exposed segment lengths for each of the five cases of PMC. ***K–L***, Nonlinear relation between the mean values of exposed segment lengths and their number. The geometrical parameters of the axons used are: axon length = 10 mm, radius = 2 μm and g-*ratio* = 0.6. The biophysical parameter values used are given in Table 1. The results shown are for *K* = 1000 independent axons for each PMC.

To characterize changes in AP conduction resulting from realistic and physiologically observed myelination motifs, we constructed nerve axon models incorporating myelin sheath heterogeneity that mirrors experimental observations (Chong et al., 2012) (see Methods). We sampled myelin sheath lengths matching those documented in the cerebral cortex and corpus callosum of mice (Chong et al., 2012) and used probabilistic tools to implement a framework that incrementally allocates these sampled sheaths to an initially fully unmyelinated (that is, bare) axon of a predetermined length (see Methods). A schematic illustrating differences between cortical and callosal myelination motifs is shown in Figure 1*A*–*B*.

To broaden the applicability of our findings to axons with different levels of myelination, we examined five distinct scenarios, each representing varying PMC for a fixed total axonal length (that is, PMC values of 10%, 30%, 50%, 70% and 90%). Figure 1*C*–*D* shows the mean lengths of myelin sheaths and exposed segments for cortical and callosal myelination motifs across all PMC scenarios considered. These statistics showcase important differences between cortical and callosal myelin sheathing: myelin sheaths in the callosum display much greater spatial heterogeneity compared to those in the cortex. Specifically, as axons traverse the cortex, myelin sheaths maintain more consistent lengths. However, in the corpus callosum, the same axons display more variable myelin sheath lengths, characterized by heavier-tailed distributions (Fig. 1*C*), aligning with experimental measurements (Chong et al., 2012). In Chong et al. (2012) and Call and Bergles (2021), it has been observed that the average lengths of myelin sheaths fall within a specific range, suggesting that differences in PMC primarily reflect variations in the number of sheaths per axon. Building upon this observation, we maintained the average length of myelin sheaths within a given range, specific to either cortical or callosal axons, while modifying their number. The resulting mean values of sheath lengths for each PMC value are illustrated in Figure 1*E* for cortical and Figure 1*F* for callosal motifs. The corresponding number of myelin sheaths is depicted in Figure 1*G*–*H*. These clearly highlight important differences in spatial heterogeneity in both myelin sheath lengths and number between cortical and callosal motifs.

Geometrically, for a given axon length, any adjustments made to the number or lengths of myelin sheaths inevitably impact the corresponding number and lengths of exposed segments. The mean lengths of exposed segments are illustrated in Figure 1*I*–*J*. For the same value of PMC, callosal motifs exhibit significantly more variable and long exposed segments compared to cortical motifs (Fig. 1*J*). One may further notice that, as the PMC increases, the lengths of exposed segments decrease: as myelin covers an increasingly large portion of the axon, exposed segments become smaller and less variable. The relationship between the mean length and number of exposed segments is plotted in Figure 1*K*–*L*. There, one can see that cortical myelination motifs (Fig. 1*K*) are characterized by a smaller number of exposed segments compared to callosal motifs (Fig. 1*L*).

To isolate the impact (if any) of myelin sheath distribution heterogeneity on AP propagation and facilitate a meaningful comparison within and between cortical and callosal motifs, we deliberately kept constant the values of axonal length, radius/caliber and myelin sheath thickness (that is, g-ratio). The biophysical parameters used (see Table 1) also remain constant. This approach introduces variability solely in the length and placement of myelin sheaths and exposed segments along the axons while ensuring that any observed differences in axonal conduction are entirely the consequence of myelin heterogeneity.

We modelled the propagation of APs along these axons using cable theory (Dayan and Abbott, 2005), adapted to account for spatially heterogeneous myelination. To simulate spike generation and conduction within exposed segments, we used a spike-conducting integrate-and-fire model (Ashida and Nogueira, 2018). The rapid spread of APs along myelin sheaths was replicated assuming pure diffusion. The resulting system of equations is further supplemented by additional conditions that account for the current flow at the paranodal junctions, where the membrane potential transitions from myelinated to exposed segments, and vice versa (see Methods).

### Influence of myelin sheath spatial heterogeneity on AP conduction

**Figure 2.**
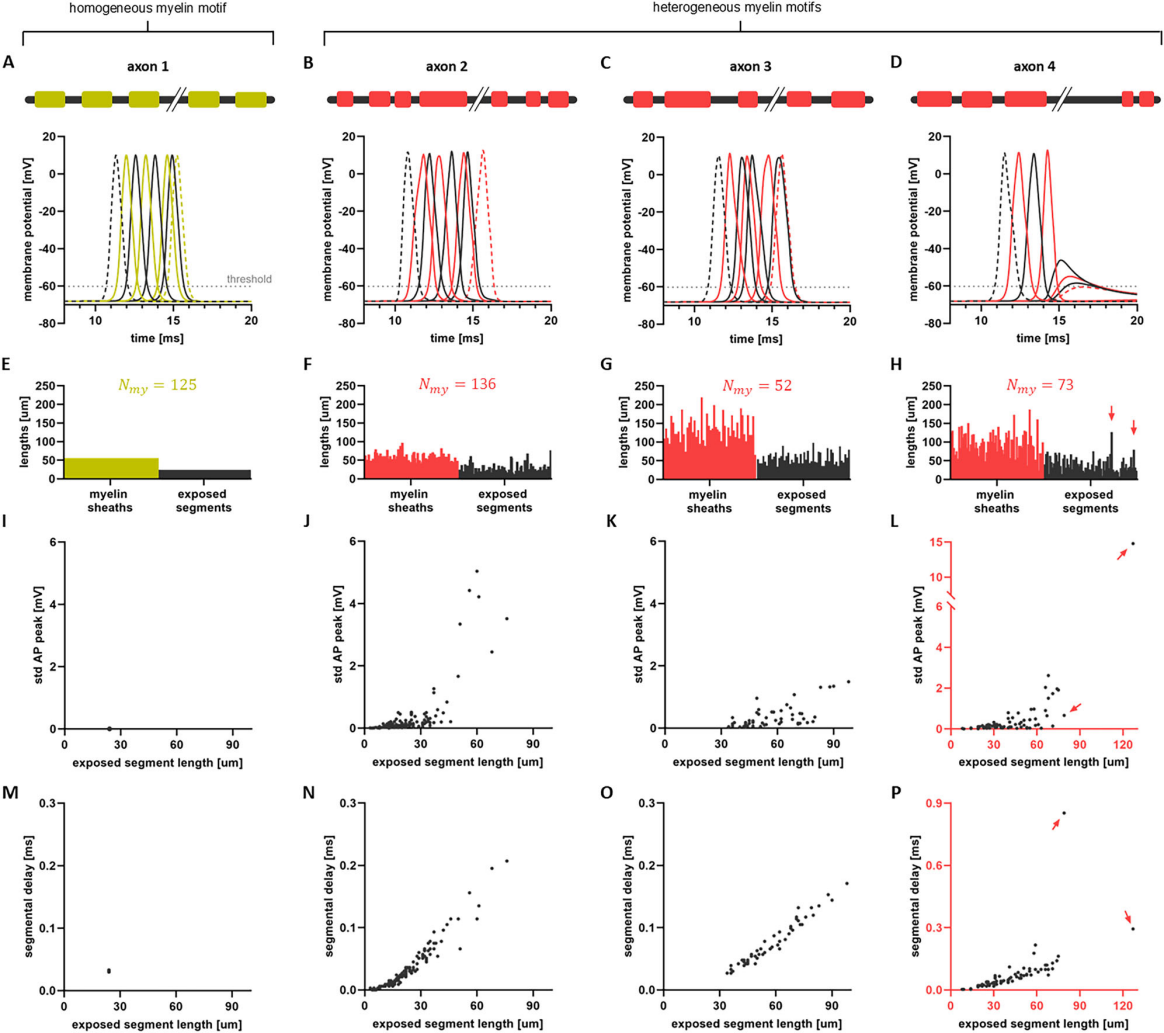
Exemplar AP propagation along axons with spatially homogeneous and heterogeneous myelin sheathing. ***A***, Snapshots illustrating the propagation of AP along an axon characterized by homogeneous myelination. ***B–D***, Propagation of APs along axons exhibiting heterogeneous myelin motifs. For all four examples, the axonal length is *L* = 10 mm and PMC is 70%. A schematic representation of each axon is displayed at the top of these panels. ***E–H***, Bar charts of the lengths of myelin sheaths and exposed segments for the four individual axons examined. Each bar in panels ***F–H*** represents the length of either a myelin sheath (red) or an exposed segment (black). As there is no variability in the lengths of sheaths and exposed segments along axons of homogeneous myelination, all those values are constant (panel *E*). The number of myelin sheaths varies for each axon and is denoted by *N*_*my*_. The number of exposed segments (*N*_*exp*_) equals the number of myelin sheaths. In panel *E*, where the length of myelin sheaths is 56 μm, resulting in a total of 125 sheaths, assuming a uniform distribution of exposed segments, their individual length is 24 μm. Note, however, that the number and length of myelin sheaths and exposed segments vary across all three axons exhibiting heterogeneous myelin motifs. ***I-L***, The standard deviation (std) of AP peaks along exposed segments is plotted as a function of the lengths of the exposed segments. In the homogeneous case (panel *I*), the variability of AP peaks is negligible compared to heterogeneous cases. In the heterogeneous cases (panels *J–L*), variability of AP peaks increases with respect to the lengths of exposed segments in a nonlinear way. ***M-P***, The delays of APs along individual exposed segments are plotted as a function of the length of those segments. In the homogeneous case, these values remain constant, as depicted in panel *I*, whereas in the heterogeneous cases, they exhibit great variability. In panels ***H***, ***L*** and ***P***, the red arrows highlight the largest exposed segments, which result in significant delays and ultimately lead to conduction block.

We started our analysis by exploring whether myelin sheathing heterogeneity influences AP propagation along individual axons. We first compared the conduction of APs along axons displaying a homogeneous myelin motif – characterized by myelin sheaths of uniform length periodically distributed along axons – with the conduction along axons featuring spatially heterogeneous myelin sheath distributions. For the purpose of this experiment, axons exhibiting callosal myelination motifs were used. Snapshots capturing the membrane potential’s evolution over time, accompanied by schematic representations of the axons, are illustrated in Figure 2*A*–*D*. Figure 2*E*–*H* present bar plots detailing the lengths of myelinated and exposed segments for each of the examined axons. For the axon with homogeneous myelination, both the lengths of myelin sheaths and exposed segments remain constant. Conversely, for the axons exhibiting heterogeneous myelination, one can see an important dispersion in those lengths. Differences could also be observed while comparing changes in APs waveform as they propagate along axons of either homogeneous or heterogeneous motifs. We characterized changes in AP waveform by quantifying the variability (i.e., standard deviation) of AP peak amplitudes computed between successive exposed segments of the axon. Specifically, we computed the AP peak within each discretized compartment of an exposed segment and calculated the standard deviation among those values. We repeated this process for each exposed segment along the axons. In the homogeneous case (Fig. 2*A*), the AP waveform remains unchanged as it propagates along the axon. This is corroborated by the near-zero variability in AP peak amplitude, as shown in Figure 2*I*. In contrast, heterogeneous myelination (Fig. 2*B*–*D*) introduces fluctuations in both the AP waveform and conduction time. Such variations in AP waveform are quantified in Figure 2*J*–*L*, where the standard deviation of AP peak amplitude along exposed segments is consistently observed, further varying along the same axon and exhibiting differing slopes across axons. These findings revealed that the particular location of myelin sheaths and exposed segments as well as their number and length lead to not only changes in AP waveform, but also instances of faster or slower conduction, as well as conduction failure (Fig. 2*D*).

These observations suggest that spatial heterogeneity of myelin sheaths and exposed segments influences axonal conduction delays. Indeed, segmental delays along exposed segments – that is the time required for an AP to traverse individual segments (see Methods) – were found to vary with the length of the segments in axons of heterogeneous motifs (axons 2, 3 and 4 in Figure 2*N*–*P*), while they remained constant for the axon of homogeneous myelination (axon 1 in Fig. 2*M*). The differing slopes in Figure 2*N*–*P* reflect the variations observed in the corresponding variability in AP waveform (Fig. 2*J*–*L*). Notably, the AP traverses short exposed segments rapidly and without significant waveform change, whereas, in longer segments, slower propagation is accompanied by significant changes in the AP peak amplitude. Additionally, APs propagated rapidly along myelinated regions, resulting in negligible delays and preserving the AP waveform (result not shown).

**Figure 3.**
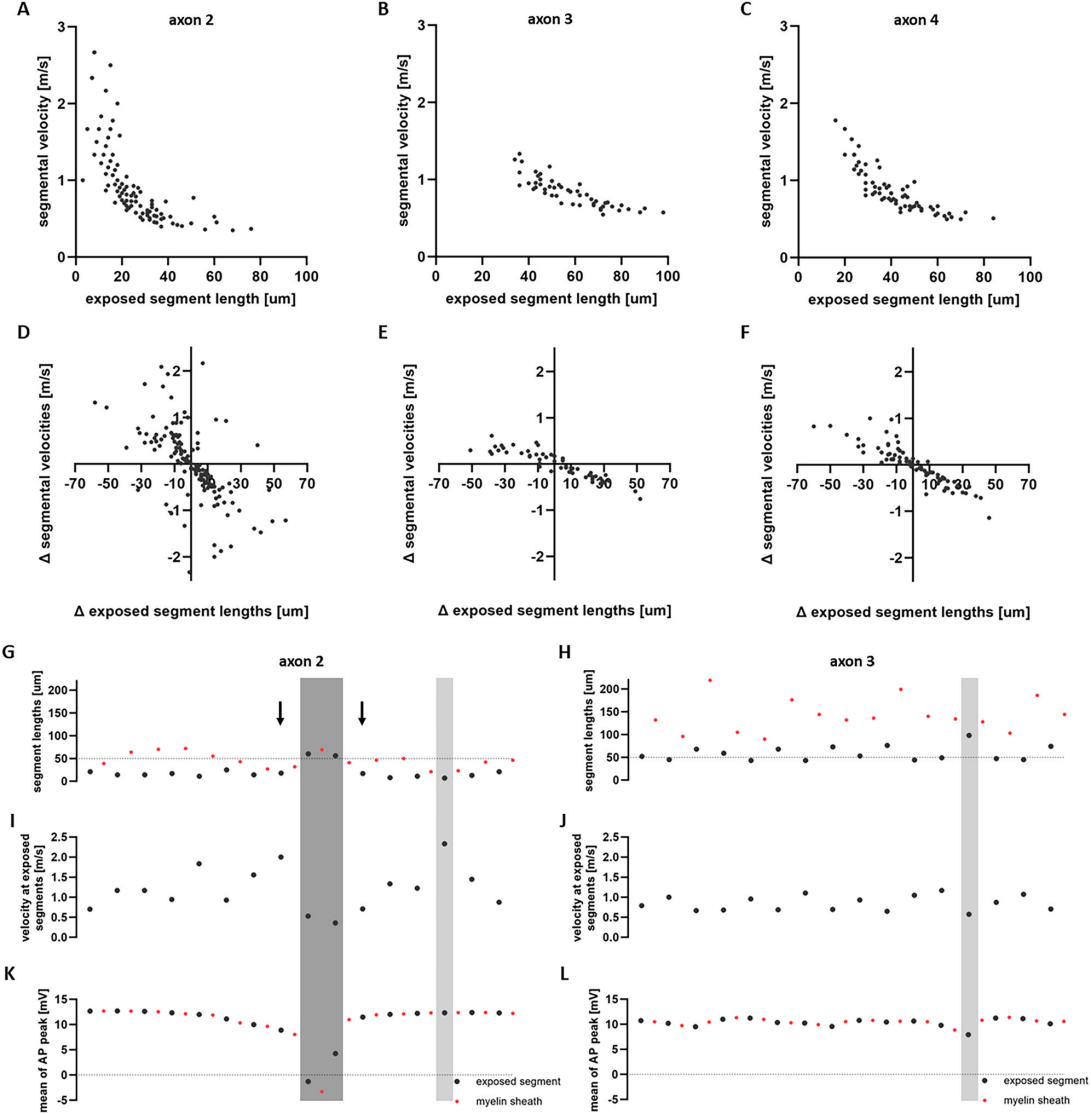
Implications of myelin sheathing heterogeneity on axonal CV along exposed segments. ***A*–*C***, AP velocities along exposed segments plotted with respect to the length of the segments for axons 2, 3 and 4 (of Fig. 2), respectively. The velocities decrease nonlinearly as the length of exposed segments increases. The slope is different for each of the three axons indicating the different dynamics the APs undergo when traveling along axons of heterogeneous myelin motifs. ***D–F***, Change (denoted by Δ) of exposed segment lengths plotted as a function of the change in velocities along exposed segments for axons 2, 3 and 4. Despite the different segment lengths encountered between the three axons, the Δ of exposed segment lengths lies in the same range. Variability of Δ in segmental velocities is larger in axon 2 compared to axons 3 and 4. ***G–H***, Lengths of myelinated and unmyelinated segments plotted in the order they appear along a section of axon 2 and axon 3. The lengths of sheaths and exposed segments are on average longer for axon 3 compared to axon 2. ***I–J***, Velocities along the corresponding exposed segments of axons 2 and 3. Along axon 2, velocities are highly variable. ***K–L***, Mean values of AP peaks along the myelin sheaths and exposed segments for axons 2 and 3. A sharp decrease of the AP peak in axon 2 occurs when the AP travels from a short segment to a much longer one. The dark grey shaded area highlights the two longest exposed segments that affect both the velocity and the mean of AP peaks. The light grey shaded areas and the arrows highlight other points of interest along the axons.

An important question remains: does the variability in AP propagation, as depicted in Figure 2, arise solely from the variability in segment lengths, or is this process also influenced by interactions between the AP dynamics in neighboring segments? To address this, we performed a detailed analysis of the conduction velocity (CV) in exposed segments, and explored whether AP conduction depends on neighboring segments. In the three axons with heterogeneous myelination shown in Figure 2 (axons 2, 3, and 4), segmental velocities decrease with exposed segment length (Fig. 3*A*–*C*). To better understand how CV is impacted by transitions from segment to segment, we calculated the change in segmental velocities, defined as the difference in CV between successive exposed segments. These changes were then plotted against the corresponding changes in exposed segment lengths (Fig. 3*D*–*F*). We denote the change by Δ. A positive Δ in exposed segment lengths indicates that a long segment is preceded by a shorter one, and vice versa. As shown in Figure 3*D*–*F*, the AP velocity increases (Δ of segmental velocities is positive) when transitioning from a long segment to a shorter one (Δ of exposed segment lengths is negative). Conversely, the AP velocity decreases (Δ of segmental velocities is negative) when transitioning from a short segment to a longer one (Δ of exposed segment lengths is positive).

In Figure 3*G*–*L*, we analyzed selected sections of axons 2 and 3 from Figure 2. We plotted the segment lengths (both myelinated and unmyelinated) in the order they appear along the axons (Fig. 3*G*–*H*), the velocity along the exposed segments (Fig. 3*I*–*J*), and the mean value of the AP peaks for myelin sheaths and exposed segments (Fig. 3*K*–*L*). For axon 2, within the dark grey shaded area, we identified two exposed segments that are noticeably longer than the other exposed segments found within the same section of the axon. This caused a sharp decrease in the AP peak amplitude and segmental velocity. The subsequent second-longest exposed segment further slowed the velocity, while the AP peak amplitude remained low but showed an upward trend. The myelin sheath between the two long exposed segments played a critical role in preventing conduction failure. Without this myelin sheath, the AP would encounter a very long exposed segment, potentially leading to conduction failure (as in axon 4, Fig. 2). An interesting observation is evident for the exposed segments immediately before and after those in the dark grey shaded area (marked by black arrows). While these two segments have approximately the same length, the velocities differ significantly. This demonstrates that the velocity along an exposed segment is influenced not only by its length but also by the dynamics along neighboring segments. The exposed segment within the light grey shaded area is the shortest in the entire section. The AP traverses this segment quickly, maintaining its amplitude. For axon 3, both the myelinated and unmyelinated segments are, on average, longer than those in axon 2. This results in less variation in velocities along the exposed segments and smaller changes in the AP peak amplitudes. Taken together these insights hint at a nonlinear relationship between the axonal conduction delay and velocity and the spatial heterogeneity of myelin sheathing.

### Heterogeneity of myelin sheathing impacts AP conduction reliability and susceptibility to failure

In light of the above observations, a natural question arises as to how spatial heterogeneity of myelin sheaths influences the reliability, propagation and efficacy of AP conduction. The distinct fluctuations of AP waveforms depicted in Figure 2*J*–*L* suggest that the time required for an AP to traverse axons is likely contingent upon the precise arrangement and lengths of myelin sheaths and exposed segments. To quantify this path-dependence, we calculated the conduction delays and the corresponding CVs of axons with identical biophysical properties, for a fixed axonal length and for each value of PMC. The lengths and number of myelin sheaths along these axons were randomly sampled from either cortical or callosal myelination motifs (see Fig. 1).

**Figure 4.**
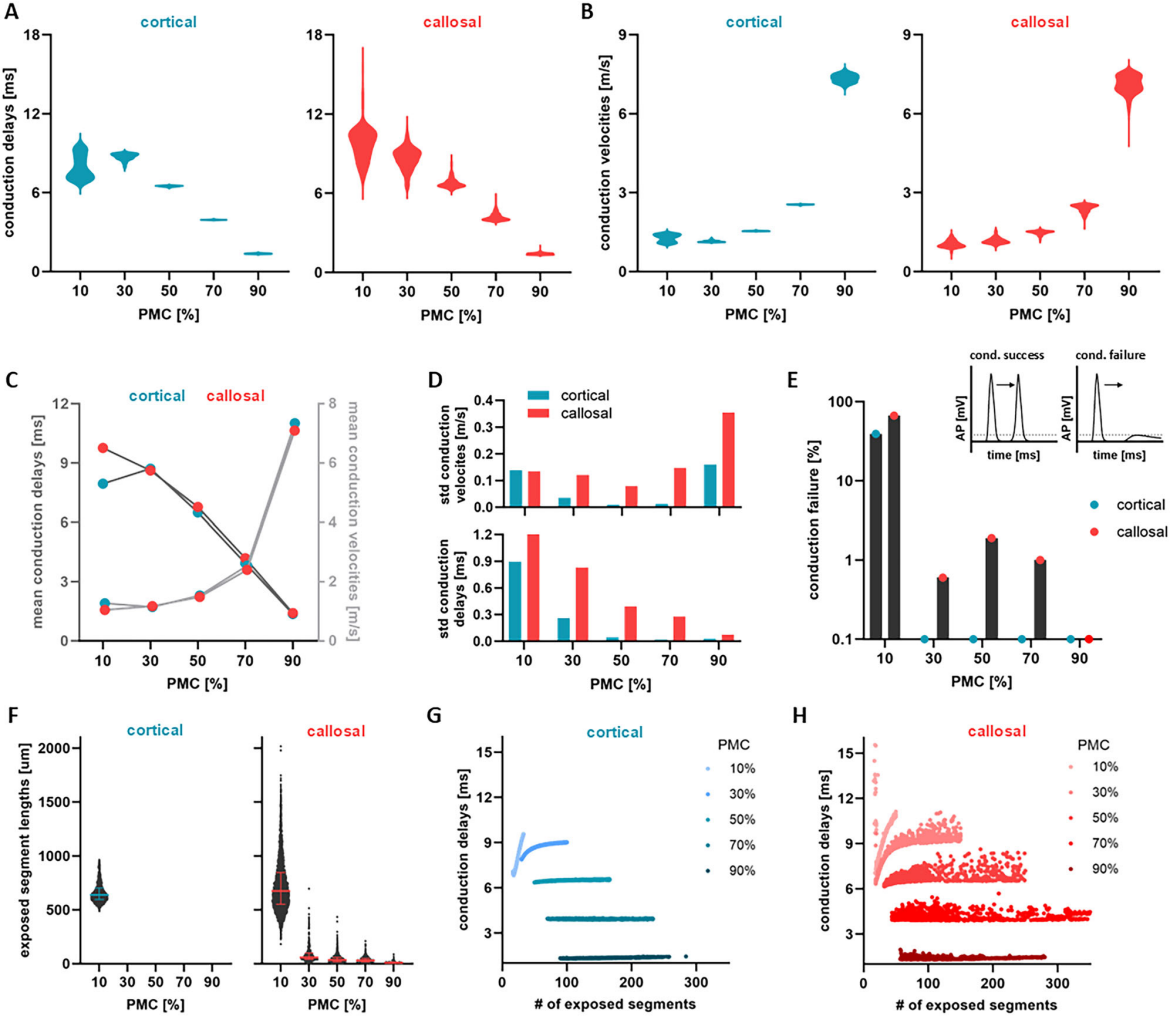
Axonal conduction variability as a result of spatially heterogeneous myelination along individual axons. ***A***, Violin plots showing the distribution of axonal conduction delays (measured in ms) plotted for different myelin coverage marking the variability in axonal conduction caused by cortical and callosal myelin motifs. ***B***, Violin plots of the corresponding distributions of axonal CVs (measured in m/s) for all five cases of PMC and for cortical and callosal myelin motifs. ***C***, Mean values of axonal conduction delays (dark grey) and CVs (light gray) as a function of PMC. ***D***, Bar plots of the standard deviation of axonal conduction delays (bottom) and axonal conduction velocities (top), for both cortical and callosal myelin motifs as a function of PMC. ***E***, Rate of axonal conduction failures, that is, the number of failures over the total number of trials, for each PMC value. The top left intake plot is a representation of conduction success. The peak of the AP remains above the threshold at all times indicating that the AP reaches successfully the end-point of the axon. The top right intake plot is a representation of a conduction failure. The peak of the AP reaches a sub-threshold value making it unable to elicit a depolarization afterwards. ***F***, Lengths of exposed segments in axons where we observe conduction failure. The left panel contains values of cortical axons and the right of callosal axons. For the cortical motifs, there are no values for PMC=30%,50%,70%and90% as zero conduction failures were observed (cf panel *E*). The thick lines in the middle correspond to the median, while the thin lines refer to the first and third quartiles. ***G–H***, Scatter plots of axonal conduction delays with respect to the number of exposed segments. In panel ***G***, the different shades of blue point out the myelin intensity along axons with cortical myelin motifs, while in panel ***H***, the different shades of red indicate myelin intensity along axons with callosal motifs. The results shown are for *K* = 1000 independent axons for each PMC.

As expected, axonal conduction delays decreased with the addition of myelin (Fig. 4*A*), while axonal CVs increased (Fig. 4*B*). While the mean axonal CVs and delays were found to be comparable between cortical and callosal myelination motifs (Fig. 4*C*), the dispersion in axonal conduction delays and velocities did exhibit important differences, indicative of variability in AP propagation between and even within cortical and callosal myelination motifs. To quantify this variability, we computed the standard deviation of axonal conduction delays and velocities. We found that conduction delay variability was larger for callosal compared to cortical motifs, a trend that persisted across all PMC scenarios considered (bottom panel of Fig. 4*D*), despite being controlled for axonal length, PMC, and other physiological parameters (see Methods; Table 1). Furthermore, we observed a negative correlation between delay variability and PMC: as PMC increases, the dispersion of axonal conduction delays decreases. The associated axonal CVs also exhibited markedly lower variability in cortical compared to callosal motifs, whose CV was more variable across PMC values (top panel of Fig. 4*D*). Variability in conduction delays at low PMC values is also associated with variability in the number of exposed segments: as the number of exposed segments increases, the dispersion in conduction delays decreases (Fig. 4*G*–*H*). Consistent with the findings above, cortical motifs demonstrated smaller variability compared to the corpus callosum. Taken together, these results confirm that regional differences in myelination significantly influence AP propagation variability.

The consequences of myelination motifs extend beyond issues of conduction time reliability. Our analysis reveals that the faithful transmission of APs (that is, conduction success or failure) is also dependent on the distribution of myelin. Conduction failure can arise due to factors such as high stimulation frequency and/or reduced capacity of membrane potentials to depolarize in regions deprived of myelin (Schauf and Davis, 1974). This has important functional implications, including the inability to elicit depolarization in postsynaptic cells. In our model, conduction failures occur when the APs fail to reach the paranodal junction while traveling along longer exposed segments (see Methods). As a result, the amplitude of the AP falls below the threshold necessary for spike initiation, as depicted in the embedded plot of Figure 4*E*. An illustration of successful conduction is portrayed in Figure 4*E*, where the AP amplitude maintains supra-threshold values throughout the entire axon. By calculating the rate of conduction failures (see Methods), we found that both cortical and callosal motifs exhibited systematic failure in cases of low myelination (PMC=10%, Figure 4*E*). Figure 4*F* shows the lengths of exposed segments in axons where conduction failure occurs. These results clearly indicate that long exposed segments are complicit to AP conduction failure, and are especially salient in the callosal motifs. These segments provide more opportunities for ion leakage across the axonal membrane, which, among other factors, contributes to the gradual decrease in depolarization amplitude necessary for AP propagation. A considerably lower rate of conduction failures was observed in axons exhibiting higher degrees of myelination, with myelination motifs inherent to the corpus callosum showing slightly greater susceptibility to failures. Such differences can be linked to the larger representation of long unmyelinated segments in callosal motifs (Fig. 1*J*). In summary, these findings suggest that failure susceptibility might increase as axons traverse the corpus callosum, while grey matter myelination motifs – inherently less heterogeneous – may demonstrate heightened resilience to changes in PMC.

### AP propagation is non-linear along heterogeneously myelinated axons

**Figure 5.**
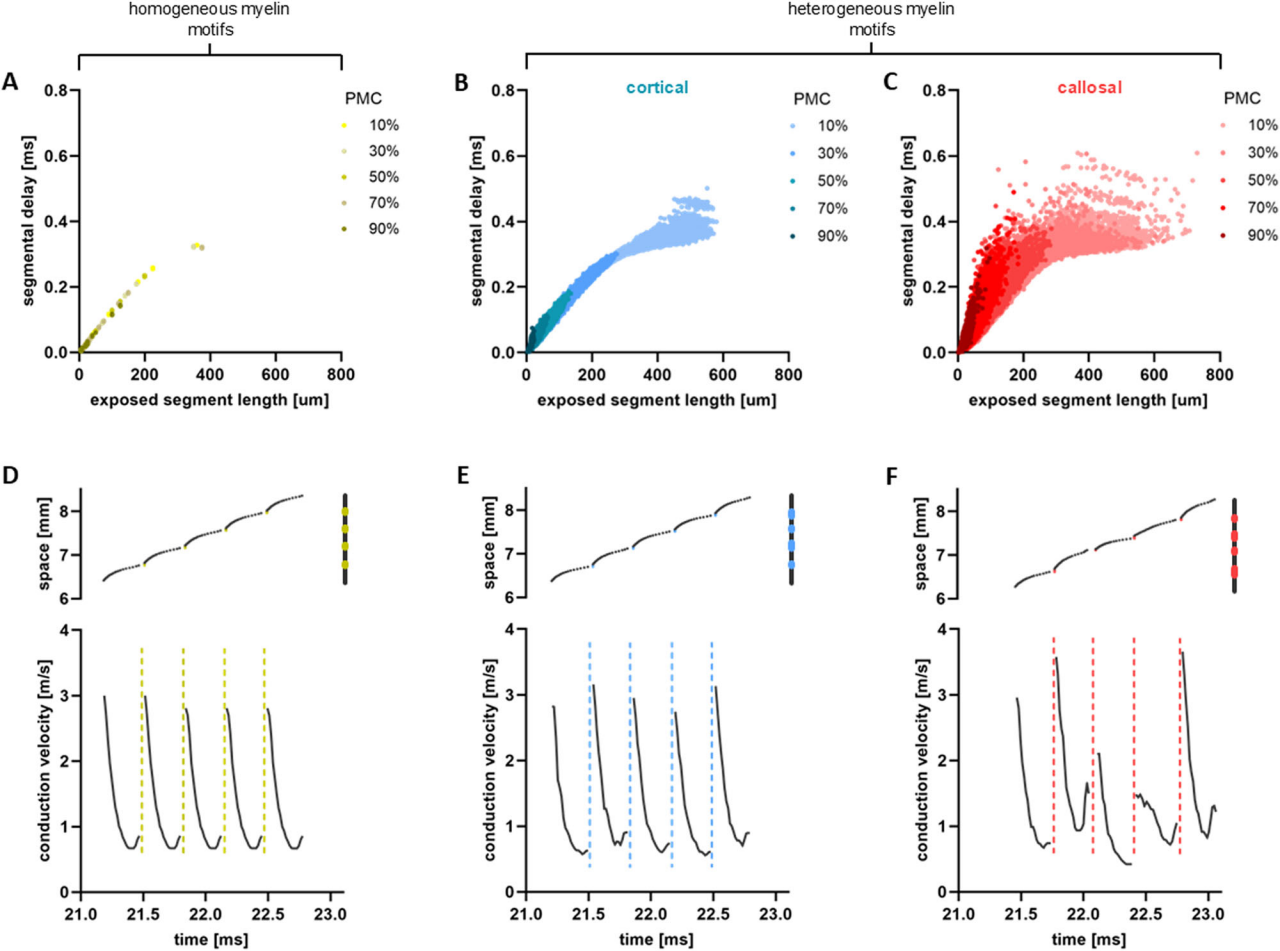
Non-linearity and path-dependence of AP propagation along axons with heterogeneous myelin sheathing. ***A–C***, Conduction delays along individual exposed segments (that is, segmental delays) are plotted as a function of their individual lengths. In panel ***A***, the model axons feature homogeneous myelin sheathing, while in panels *B* (blue; cortical) and *C* (red; callosal) myelin sheaths are spatially heterogeneous. In each panel, color shading indicates PMC: light colors refer to low PMC values, and darker colors indicate high PMC values. The plotted data are for *K* = 1000 independent axons for each of the myelination motifs and PMC. ***D***, (top panel) For a section of an individual axon with homogeneous myelination, we plotted how the propagation time of AP changes in space (that is, along successive segments of the axon). The black dots represent the time within exposed segments and the yellow dots the time within myelin sheaths. On the right side of the plot is a schematic representation of the section of the axon. (bottom panel) Corresponding CVs within each of the segments. The dashed yellow lines represent the asymptotic behavior of the CVs within myelin sheaths. ***E–F***, Similarly, for an axon with cortical (panel *E*) and callosal (panel *F*) myelination motif. For panels ***D–F***, simulations are for individual axons of length *L* =10 mm, PMC=10% and *N*_*my*_ = 25. Biophysical parameter values can be found in Table 1.

The results depicted in Figures 2–4 suggest that axonal conduction delays are not solely determined by PMC or total axonal length; rather, conduction timing depends on the specific placement, ordering, and lengths of myelin sheaths and exposed segments. Indeed, even when controlling for differences in PMC and axonal length, conduction delays demonstrate important variability directly attributable to cortical or callosal myelin sheath arrangement (Fig. 4*D*). In Figure 5*A*–*C*, we plotted segmental delays against the length of the corresponding segment. Given that APs propagate rapidly along myelin sheaths through diffusion (see Methods), such delays are predominantly due to propagation along exposed segments. For axons with homogeneous myelin sheathing, conduction time scales linearly with the lengths of exposed segments, except for very long segments (Fig. 5*A*). The dispersion of segmental delays is zero, and the CV, reflected in the slope of the curve, remains constant. In contrast, axons with cortical and callosal motifs show increased segmental delay dispersion proportional to myelin sheath heterogeneity and PMC. The CVs fluctuate across segments and axons, as evidenced by the important variability in the slopes of the curves in Figure 5*B*–*C*. In line with our previous observations, callosal motifs did exhibit a more salient non-linearity combined with heightened variability of segmental delays, a feature directly linked to the statistical differences in exposed/myelinated segment lengths and number as well as the positioning of the segments on axons, compared to the cortical motifs (Fig. 1). These findings propose that axonal conduction is a path-dependent process, sensitive to cumulative transitions between myelinated and exposed segments of the axon, thereby confirming a dependence of AP propagation on myelination motifs.

To better understand this nonlinear behavior, we examined in further detail AP propagation along individual segments (Fig. 5*D*–*F*). Along segments covered by myelin, transmission occurs instantaneously, as expected from saltatory conduction, while across exposed segments transmission slows down due to ionic currents (see Methods), leading to a nonlinear increase in AP propagation time. In a homogeneously myelinated axon, AP propagation time follows a periodic pattern across segments, repeated throughout the entire axon (Fig. 5*D* – top panel). However, for heterogeneously myelinated axons, delays were found to increase irregularly along successive exposed segments, with more pronounced changes observed for the callosal motif, which we recall is more heterogeneous (Fig. 5*E*–*F* – top panels). These trends are further reflected in the corresponding changes in CV experienced by the AP as it propagates along individual segments. Along myelin sheaths, CV is extremely high, reflecting the instantaneous spike timing. In contrast, within exposed segments, CV rapidly decreases as the AP enters the segments, and slightly increases before reaching the junction of the next myelin sheath (Fig. 5*D*–*F* – bottom panels). Mirroring the trend seen for the conduction delay, CV follows a periodic pattern in the homogeneously myelinated axon (Fig. 5*D*). However, fluctuations in CV were found to be especially irregular for the callosal motif (Fig. 5*F*). Taken together, these results suggest that APs are subjected to bouts of deceleration and acceleration along exposed segments. These collectively result in a deeply non-linear, path-dependent process, by which AP conduction becomes highly sensitive to the dynamics between neighboring segments (i.e., see Fig. 3), and thus myelin sheathing heterogeneity. It is interesting to consider the potential implications of the observed non-linearity in conduction delays and velocities, for instance when myelination motifs undergo demyelination.

### Vulnerability of heterogeneous myelination motifs to demyelination

**Figure 6.**
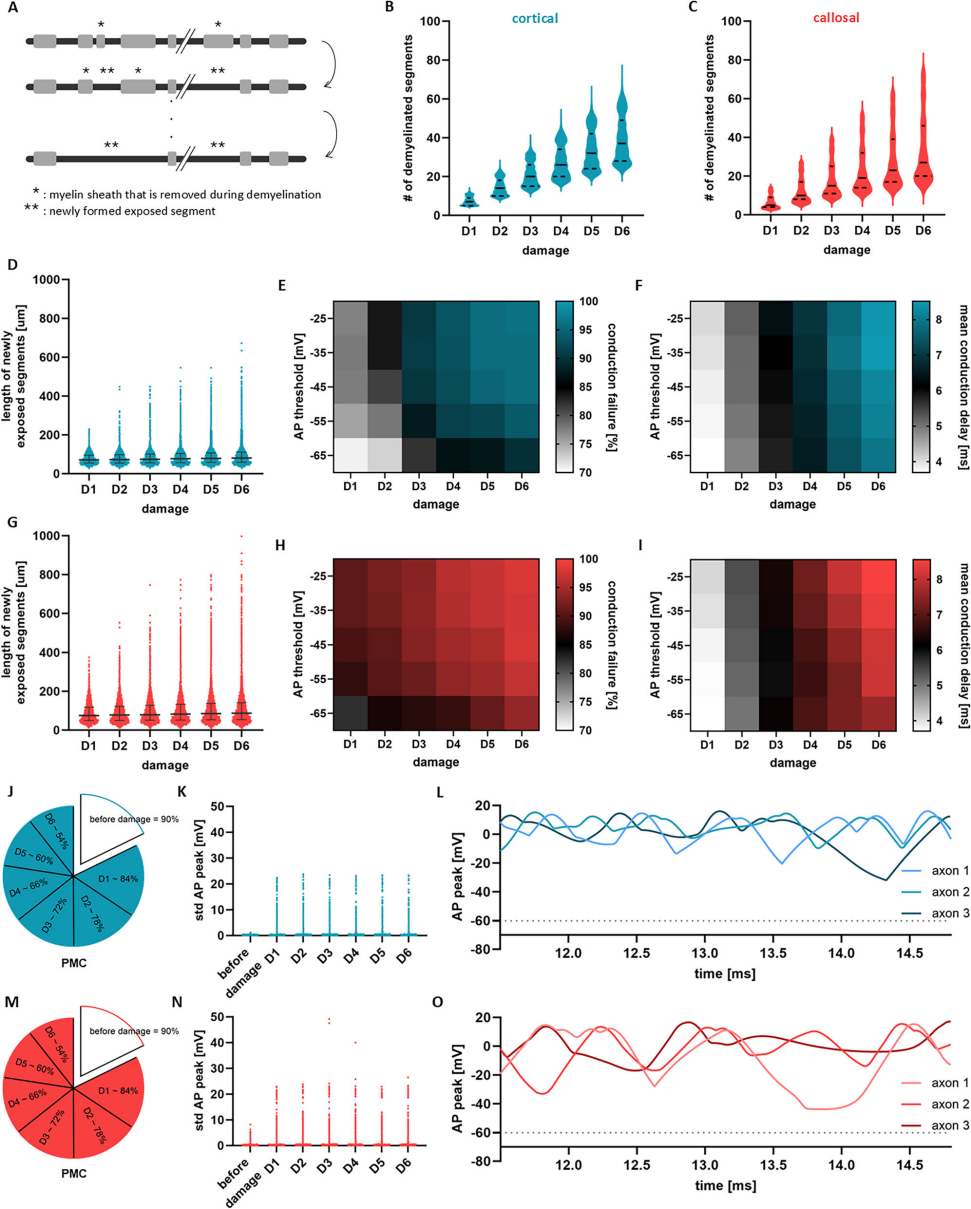
Vulnerability of cortical and callosal motifs to myelin injury. ***A***, Schematic of the demyelination process. Axons of length *L* = 10 mm and initial PMC=90% undergo successive myelin removal, in which the number of myelin sheaths is reduced by 5% each time. After demyelination, longer exposed segments are formed. This process is repeated six times to model different levels of severity of myelin damage (D1, …, D6). ***B–C***, Scatter plots of the number of demyelinated segments along axons for all six steps of myelin damage for cortical (panel *B*) and callosal (panel *C*) myelination motifs. The middle lines in the violin plots indicate the median and the lower and upper dashed lines indicate the first and third quartiles, respectively. ***D***, Scatter plot of the length of exposed segments after demyelination (for cortical axons). These include only the segments directly affected by demyelination. The thick lines in the middle correspond to the median while the thin lines to the quartiles. ***E***, Heat map of the rate of conduction failure as a function of AP threshold and for each step of myelin damage for axons with cortical myelin motifs. ***F***, Mean values of axonal conduction delays (only for axons with cortical motifs that successfully conduct) as a function of AP threshold and degree of myelin damage. ***G–I***, Corresponding plots of newly formed exposed segment lengths, rate of conduction failure and mean of axonal conduction delays for axons with callosal myelin motifs. ***J***, Pie chart showing the average PMC per demyelination stage for cortical motifs. ***K***, Standard deviation of AP peaks along the exposed segments of cortical axons before and after damage. The data collected only for axons of successful conduction. ***L***, Representative examples of the evolution of AP for three cortical axons after demyelination. The dotted line denotes the AP threshold. ***M–O***, Corresponding plots of the PMC, the standard deviation of AP peaks and examples of AP peak as a function of time for axons with callosal myelin motifs. The results shown are for *K* = 500 independent axons for each stage of demyelination.

Upon the onset of demyelination, the integrity of myelin sheaths becomes compromised or completely breaks down, subsequently causing disruptions in the transmission of nerve impulses (Schauf and Davis, 1974). Such demyelination results from injury or loss of myelin sheaths and/or OLs, and occurs in numerous neurological (Duncan and Radcliff, 2016) and neuropsychiatric (Fields, 2008; Takahashi et al., 2011) disorders. Given the statistical differences in lengths, number and spatial distributions of myelin sheaths (Fig. 1), is AP conduction along axons with cortical or callosal motifs equally vulnerable to demyelination? To answer this question, we examined how cortical and callosal myelination motifs were impacted by myelin injury, and quantified their resulting predisposition to conduction failure. We modeled demyelination in two steps: (i) by systematically reducing the number of myelin sheaths, and (ii) by varying the excitability of the affected areas, specifically the demyelinated segment and the nodes adjacent to it. In other words, we varied the AP threshold along the newly formed exposed segments, while maintaining the baseline threshold value (−60.2 mV) in the unaffected segments. This was done to account for a pathological decrease in ion channel density (see Methods). A schematic illustration portraying the demyelination process described above is presented in Figure 6*A*. The reduction in the number of myelin sheaths naturally affects the PMC in damaged axons. Specifically, we initially set the PMC for axons before demyelination at 90%, while 5% of the total number of myelin sheaths were successively removed at each iteration. Through this process, the number of demyelinated segments gradually increases (Fig. 6*B*–*C*). Demyelinated segments along callosal axons expand to cover a broader range compared to cortical axons, as a consequence of the difference in distribution prior to demyelination (Fig. 1*G*–*H*). Figure 6*D*,*G* illustrates the lengths of the newly exposed segments, with callosal motifs demonstrating both much shorter and much longer segments compared to cortical motifs.

To measure vulnerability, we computed the rate of axonal conduction failures for various levels of excitability (i.e., AP threshold values, that change only along the newly exposed segments) and for successive application of myelin damage (Fig. 6*E*,*H*). We observed that callosal motifs were more vulnerable to demyelination, compared to cortical ones. This is confirmed by the higher rates of conduction failure (Fig. 6*H*). For both cortical and callosal motifs, conduction failures were expectedly found to be more prominent as the excitability of newly exposed segments decreases (that is, depolarization threshold increase, reflecting lower ion channel density). Next, we examined how demyelination affected the conduction delays along axons that did not experience failure in spite of experiencing extensive damage. Figure 6*F,I* showcases an increase in mean conduction delays as the axons become increasingly demyelinated. This trend was equally observed in both cortical and callosal motifs. At a fixed stage of demyelination, varying the AP threshold along the newly exposed segments affected only the rate of conduction failures, which increased with higher AP thresholds (Fig. 6*E,H*), while the mean conduction delays remained largely unchanged (Fig. 6*F,I*). This pattern was consistent across all stages of demyelination.

Figure 6*J,M* shows the amount of myelin coverage for each stage of demyelination. Comparing the mean conduction delays before (Fig. 4*C*) and after myelin damage (Fig. 6*F,I*) reveals a significant difference for axons with similar levels of myelin coverage. To investigate this further, we quantified the AP waveform by calculating the standard deviation of the AP peak amplitude as it propagated along the axons (Fig. 6*K*–*L* for cortical and Fig. 6*N*–*O* for callosal). Clearly, AP waveform demonstrates greater variability when traversing demyelinated axons. Such variability predisposes damaged axons to slower conduction or conduction block: demyelination-induced modulation of AP peak amplitude can be seen as interrogating both local excitability and myelin coverage, making the axon more susceptible to slow propagation or failure, particularly when the AP encounters long unmyelinated segments. We highlight that this is another salient manifestation of the path-dependent nature of axonal conduction along axons exhibiting heterogeneous myelin sheathing.

## Discussion

Numerous experimental studies have revealed that mammalian axons display a wide diversity of myelination patterns. Significant heterogeneity in the number, thickness, length, and distribution of myelin sheaths along and between different axons, as well as across brain regions (Sturrock, 1980; Bakiri et al., 2011; Glasser and Essen, 2011; Chong et al., 2012; Tomassy et al., 2014; Ford et al., 2015; Micheva et al., 2016; Auer et al., 2018; Hill et al., 2018; Stedehouder et al., 2019; Benamer et al., 2020; Call and Bergles, 2021) has been observed. Notably, axons exhibit markedly different myelin sheathing motifs as they traverse the cortex and/or corpus callosum (Geurts and Barkhof, 2008; Glasser and Essen, 2011; Chong et al., 2012; Tomassy et al., 2014; Calabrese et al., 2015; Prins et al., 2015; Micheva et al., 2016; Timmler and Simons, 2019; Orthmann-Murphy et al., 2020; Call and Bergles, 2021; Corrigan et al., 2021; Lie et al., 2022; Thornton et al., 2024). However, how this variability influences neural signaling and susceptibility to injury remains poorly understood. Spatial heterogeneity in myelin sheathing is challenging to characterize with existing computational frameworks (Fitzhugh, 1962; Goldman and Albus, 1968; Grindrod and Sleeman, 1985; Basser, 1993; Nygren and Halter, 1999; McIntyre et al., 2002; Gow and Devaux, 2008; Babbs and Shi, 2013; Ashida and Nogueira, 2018; Scurfield and Latimer, 2018; Naud and Longtin, 2019; Schmidt and Knösche, 2019), often considering the axon as a cable with uniform myelin sheathing interspersed with exceedingly brief nodes of Ranvier. Motivated by the critical role played by myelin on AP conduction and its well-documented consequence on brain function (Nagy et al., 2004; Bengtsson et al., 2005; Mabbott et al., 2006; Pujol et al., 2006; McKenzie et al., 2014; Pan et al., 2020; Steadman et al., 2020; Xin and Chan, 2020), we here explored the influence of spatially heterogeneous myelin sheathing on axonal conduction reliability and predisposition to failure. Specifically, we developed a mathematical model of excitable axons displaying myelination motifs mirroring those observed experimentally in the cortex and corpus callosum of mice (Chong et al., 2012), brain areas known for their salient differences in myelin sheathing motifs (Geurts and Barkhof, 2008; Glasser and Essen, 2011; Chong et al., 2012; Tomassy et al., 2014; Calabrese et al., 2015; Prins et al., 2015; Micheva et al., 2016; Timmler and Simons, 2019; Orthmann-Murphy et al., 2020; Call and Bergles, 2021; Corrigan et al., 2021; Lie et al., 2022; Thornton et al., 2024). Leveraging experimental data (Chong et al., 2012), we systematically compared how such myelination motifs influence AP conduction delays, CVs as well as vulnerability to failure in these areas, for both healthy axons and those subjected to demyelination.

Our analysis revealed that variability in myelin patterns has an important impact on the reliability of axonal conduction. Indeed, axonal conduction was confirmed to be a nonlinear, path-dependent process, thereby sensitive to myelin sheath arrangement through the cumulative effect of AP propagating across myelinated and/or exposed segments of various lengths. Variability in conduction delays was found to correlate inversely with myelin coverage and differed between callosal and cortical motifs. This variability was however found to be more prominent amongst callosal motifs. We emulated demyelinating damage by selectively removing sheaths and quantifying resulting changes in AP conduction. Callosal myelination motifs, in particular, were found to display a greater sensitivity to demyelination, exhibiting increased failure rates compared to cortical motifs.

The diversity in myelin sheaths distribution along axons is the manifestation of highly plastic, area- and cell-type specific myelination processes, as well as the consolidation of signaling pathways by OLs. Through adaptive myelination and remodeling, myelin sheaths may retract and/or elongate (Gibson et al., 2014; Baraban et al., 2018; Krasnow et al., 2018), altering the lengths of exposed segments/nodes of Ranvier in an axon-specific manner (Arancibia-Cárcamo et al., 2017; Koskinen et al., 2023), thereby resulting in spatially heterogeneous myelination pattern. The manifest differences in myelin motifs (Chong et al., 2012; Tomassy et al., 2014; Timmler and Simons, 2019) and plasticity (Thornton et al., 2024) observed between grey and white matter axons suggest that precise sheath placement plays an important functional role (Timmler and Simons, 2019; Orthmann-Murphy et al., 2020). Myelin integrity has indeed been linked to a wide array of brain functions ranging from memory (Pan et al., 2020; Steadman et al., 2020) and learning (Bengtsson et al., 2005; McKenzie et al., 2014; Xin and Chan, 2020; Bacmeister et al., 2022) to executive functions (Nagy et al., 2004; Mabbott et al., 2006; Pujol et al., 2006).

In contrast, compromised myelin integrity, resulting from the loss of myelin sheaths and/or OLs impairment, is linked to an increasing range of neurological (Duncan and Radcliff, 2016; de Faria Jr et al., 2021) and neuropsychiatric conditions (Fields, 2008; Takahashi et al., 2011; de Faria Jr et al., 2021). Aberrant and/or maladaptive changes such as the lengthening of the nodes of Ranvier, breakdown of the electrically insulating barrier between the myelin sheath and the axonal membrane at the paranodal region, and shrinkage of the sheaths leading to the exposure of K^+^ channels in the juctaparanodes, are also potential contributors to the modulation of myelinated axon functionality. These changes could result in a slowdown of the AP propagation or even a conduction failure across a diverse spectrum of pathological conditions (Arancibia-Carcamo and Attwell, 2014; Freeman et al., 2016; Dolma and Joshi, 2023).

In line with multiple experimental studies (Geurts and Barkhof, 2008; Calabrese et al., 2015; Prins et al., 2015; Orthmann-Murphy et al., 2020; Call and Bergles, 2021; Lie et al., 2022; Thornton et al., 2024), our results identify a significant difference between myelination motifs in the cortex and corpus callosum in response to injury, with important potential implications in myelin disorders. This difference echoes a long-standing conundrum in the literature pertaining to the pathological manifestation, consequences and clinical relevance of grey versus white matter lesions observed across multiple sclerosis stages (Geurts and Barkhof, 2008; Prins et al., 2015; Lie et al., 2022). It is interesting to conjecture that variations in myelination patterns and resulting differences in vulnerability to demyelinating damage reported here may be involved in MS progression. For instance, the relatively higher resilience of cortical myelination motifs to damage (cf. Fig. 6) allows us to hypothesize that grey matter could involved in later stages of the disease compared to the white matter. Additionally, recent findings have linked aberrant myelination of the corpus callosum (but not the cortex) and epilepsy progression in mice (Knowles et al., 2022). These results indicate that maladaptive myelination in the corpus callosum may predispose brain networks to seizures by amplifying pathological oscillations. Our predictions pertaining to the heightened sensitivity of callosal motifs to changes in myelin suggest that the corpus callosum might represent a network of high vulnerability to maladaptive changes in myelin. Further research is required to delineate the respective contributions of grey and white matter axons in severity and progression of myelin-related disorders.

Recent advances in two- and three-photon fluorescence microscopy in vivo permit the imaging of myelin sheath patterns in mammalian circuits with unprecedented resolution (Hughes et al., 2018; Orthmann-Murphy et al., 2020; Thornton et al., 2024). Longitudinal changes in myelination have further recently been linked directly with perturbations in neural activity occurring in developmental, adaptive and/or remyelinating contexts (see Hughes et al., 2018; Bacmeister et al., 2022; Osso and Hughes, 2024 and references therein). We emphasize that these experimental approaches, combined with single cell electrophysiological recordings (e.g., spike timing, interspike-interval (ISI) distributions), could be used to test our model predictions, while informing further modeling work. Such experiments could provide novel insights as to the long debated relationship between myelination sheathing motifs and neural signaling (Salami et al., 2003; Mount and Monje, 2017), essential to understand the functional implications of the aforementioned differences in myelination observed between the grey and white matter.

While providing valuable insights, our study nonetheless faces limitations that should be acknowledged. First, our modeling framework balances physiological realism and computational tractability. To face the challenge of quantifying AP conduction along thousands of axons with different myelin sheathing placement patterns, we used a phenomenological model (Ashida and Nogueira, 2018). This approach relies on stereotyped models of axonal excitability (e.g., exponential integrate-and-fire model), combined with passive diffusion through the use of the cable equation. While good correspondence between the dynamics of such simplified models and more detailed biophysical models (e.g., Arancibia-Cárcamo et al., 2017) has been reported (Ashida and Nogueira, 2018; Schmidt and Knösche, 2019), future work should examine the combined effect of explicit ionic currents and myelin sheathing heterogeneity on conduction variability.

Second, we introduced variability exclusively to the longitudinal lengths of myelin sheaths and exposed segments, while keeping the axon radius constant and refraining from altering the myelin thickness. Introducing additional sources of variability, such as the g-ratio and/or axon diameter (Talidou et al., 2021) would provide a more comprehensive understanding of how these factors interact and influence axonal conduction, and could significantly expand the scope of our conclusions.

Third, we treated exposed segments of various lengths as nodes of Ranvier, assuming that ionic conductance does not change with length. Specifically, our model did not account for the different types of ion channels, their distribution along the exposed segments, or potential variation in their density within a given axon. These factors could affect the CV and variability of signal transmission. A detailed study incorporating ion channel dynamics and their spatial distribution could address this limitation.

Lastly, in characterizing the relationship between diverse myelination motifs and variability in axonal conduction delay, we used a phenomenological approach grounded in axonal geometry. This approach assumed infinite resistance along myelin sheaths and neglected the morphological structure of the paranodes. A more physiologically detailed model, incorporating additional features at the paranodal junctions, would significantly enhance the physiological relevance of our model, and would allow us to better understand the role played by paradonal junctions in AP conduction, providing a more accurate representation of signal transmission.

## Synthesis

Reviewing Editor: Niraj Desai, National Institute of Neurological Disorders and Stroke Decisions are customarily a result of the Reviewing Editor and the peer reviewers coming together and discussing their recommendations until a consensus is reached. When revisions are invited, a fact-based synthesis statement explaining their decision and outlining what is needed to prepare a revision will be listed below. The following reviewer(s) agreed to reveal their identity: NONE.

Thank you for responding well to the reviewer comments. One reviewer still had an additional concern that you probably will have no trouble addressing. Please do so. This is a good study. Thank you for submitting it to eNeuro.

REVIEWER COMMENT

I have a minor concern regarding how the authors calculated the 'variability of AP peak amplitudes' for Fig. 2 I-L. If the authors refer to the variability as the std. dev of peak in every computational segment of each exposed segment, this is perhaps not clear to the reader. In that case, I'm not sure why the std. dev should be zero for the homogenous condition. This point requires more clarification.

## References

[B1] Arancibia-Carcamo I, Attwell D (2014) The node of Ranvier in CNS pathology. Acta Neuropathol 128:161–175. 10.1007/s00401-014-1305-z 24913350 PMC4102831

[B2] Arancibia-Cárcamo IL, Ford MC, Cossell L, Ishida K, Tohyama K, Attwell D (2017) Node of Ranvier length as a potential regulator of myelinated axon conduction speed. eLife 6:e23329. 10.7554/eLife.23329 28130923 PMC5313058

[B3] Ashida G, Nogueira W (2018) Spike-conducting integrate-and-fire model. eNeuro 5:ENEURO.0112-18.2018. 10.1523/ENEURO.0112-18.2018 30225348 PMC6140110

[B4] Auer F, Vagionitis S, Czopka T (2018) Evidence for myelin sheath remodeling in the CNS revealed by in vivo imaging. Curr Biol 28:549–559.e3. 10.1016/j.cub.2018.01.017 29429620

[B5] Babbs C. F., Shi R., Hamblin M. (2013) Subtle paranodal injury slows impulse conduction in a mathematical model of myelinated axons. PLoS ONE 8:e67767. 10.1371/journal.pone.0067767 23844090 PMC3701069

[B6] Bacmeister C, Huang R, Osso L, Thornton MA, Conant L, Chavez AR, Poleg-Polsky A, Hughes EG (2022) Motor learning drives dynamic patterns of intermittent myelination on learning-activated axons. Nat Neurosci 25:1300–1313. 10.1038/s41593-022-01169-4 36180791 PMC9651929

[B7] Bakiri Y, Káradóttir R, Cossell L, Attwell D (2011) Morphological and electrical properties of oligodendrocytes in the white matter of the corpus callosum and cerebellum. J Physiol 589:559–573. 10.1113/jphysiol.2010.201376 21098009 PMC3055543

[B8] Baraban M, Koudelka S, Lyons DA (2018) Ca^2+^ activity signatures of myelin sheath formation and growth in vivo. Nat Neurosci 21:19–23. 10.1038/s41593-017-0040-x 29230058 PMC5742537

[B9] Basser P (1993) Cable equation for a myelinated axon derived from its microstructure. Med Biol Eng Comput 31:S87–S92. 10.1007/BF02446655 8231331

[B10] Benamer N, Vidal M, Balia M, Angulo M (2020) Myelination of parvalbumin interneurons shapes the function of cortical sensory inhibitory circuits. Nat Commun 11:5151. 10.1038/s41467-020-18984-7 33051462 PMC7555533

[B11] Bengtsson S, Nagy Z, Skare Se.a. (2005) Extensive piano practicing has regionally specific effects on white matter development. Nat Neurosci 8:1148–1150. 10.1038/nn1516 16116456

[B12] Bradl M, Lassmann H (2010) Oligodendrocytes: biology and pathology. Acta Neuropathol 119:37–53. 10.1007/s00401-009-0601-5 19847447 PMC2799635

[B13] Calabrese M, Magliozzi R, Ciccarelli O, Geurts JJG, Reynolds R, Martin R (2015) Exploring the origins of grey matter damage in multiple sclerosis. Nat Rev Neurosci 16:147–158. 10.1038/nrn3900 25697158

[B14] Call C, Bergles D (2021) Cortical neurons exhibit diverse myelination patterns that scale between mouse brain regions and regenerate after demyelination. Nat Commun 12:4767. 10.1038/s41467-021-25035-2 34362912 PMC8346564

[B15] Carr E, Turner I (2016) A semi-analytical solution for multilayer diffusion in a composite medium consisting of a large number of layers. Appl Math Model 40:7034–7050. 10.1016/j.apm.2016.02.041

[B16] Chong SYC, et al. (2012) Neurite outgrowth inhibitor Nogo-A establishes spatial segregation and extent of oligodendrocyte myelination. Proc Natl Acad Sci 109:1299–1304. 10.1073/pnas.1113540109 22160722 PMC3268264

[B17] Corrigan NM, Yarnykh VL, Hippe DS, Owen JP, Huber E, Zhao TC, Kuhl PK (2021) Myelin development in cerebral gray and white matter during adolescence and late childhood. NeuroImage 227:117678. Published under a Creative Commons license 10.1016/j.neuroimage.2020.117678 33359342 PMC8214999

[B18] Dayan P, Abbott L (2005) *Theoretical neuroscience*. Cambdridge: MIT Press

[B19] de Faria Jr O, Pivonkova H, Varga B, Timmler S, Evans KA, Káradóttir RT (2021) Periods of synchronized myelin changes shape brain function and plasticity. Nat Neurosci 24:1508–1521. 10.1038/s41593-021-00917-2 34711959

[B20] Dolma S, Joshi A (2023) The node of Ranvier as an interface for Axo–Glial interactions: perturbation of Axo–Glial interactions in various neurological disorders. J Neuroimmune Pharmacol 18:215–234. 10.1007/s11481-023-10072-z 37285016

[B21] Duncan ID, Radcliff AB (2016) Inherited and acquired disorders of myelin: the underlying myelin pathology. Exp Neurol 283:452–475. 10.1016/j.expneurol.2016.04.002 27068622 PMC5010953

[B22] Fields RD (2008) White matter in learning, cognition and psychiatric disorders. Trends Neurosci 31:361–370. 10.1016/j.tins.2008.04.001 18538868 PMC2486416

[B23] Fitzhugh R (1962) Computation of impulse initiation and saltatory conduction in a myelinated nerve fiber. Biophys J 2:11–21. 10.1016/S0006-3495(62)86837-4 13893367 PMC1366385

[B24] Ford M, Alexandrova O, Cossell L, Stange-Marten A, Sinclair J, Kopp-Scheinpflug C, Pecka M, Attwell D, Grothe B (2015) Tuning of Ranvier node and internode properties in myelinated axons to adjust action potential timing. Nat Commun 6:8073. 10.1038/ncomms9073 26305015 PMC4560803

[B25] Freeman S, Desmazières A, Fricker D, Lubetzki C, Sol-Foulon N (2016) Mechanisms of sodium channel clustering and its influence on axonal impulse conduction. Cell Mol Life Sci 73:723–735. 10.1007/s00018-015-2081-1 26514731 PMC4735253

[B26] Geurts JJ, Barkhof F (2008) Grey matter pathology in multiple sclerosis. Lancet Neurol 7:841–851. 10.1016/S1474-4422(08)70191-1 18703006

[B27] Gibson EM, et al. (2014) Neuronal activity promotes oligodendrogenesis and adaptive myelination in the mammalian brain. Science 344:1252304. 10.1126/science.1252304 24727982 PMC4096908

[B28] Glasser MF, Essen DCV (2011) Mapping human cortical areas in vivo based on myelin content as revealed by T1- and T2-weighted MRI. J Neurosci 31:11597–11616. Cover Article, Behavioral/Systems/Cognitive 10.1523/JNEUROSCI.2180-11.2011 21832190 PMC3167149

[B29] Goldman L, Albus JS (1968) Computation of impulse conduction in myelinated fibers; theoretical basis of the velocity-diameter relation. Biophys J 8:596–607. 10.1016/S0006-3495(68)86510-5 5699798 PMC1367402

[B30] Gow A, Devaux J (2008) A model of tight junction function in central nervous system myelinated axons. Neuron Glia Biol 4:307–317. 10.1017/S1740925X09990391 20102674 PMC2957896

[B31] Grindrod P, Sleeman B (1985) A model of a myelinated nerve axon: threshold behaviour and propagation. J Math Biol 23:119–135. 10.1007/BF00276561 4078496

[B32] Hickson R, Barry S, Mercer G, Sidhu H (2011) Finite difference schemes for multilayer diffusion. Math Comput Model 54:210–220. 10.1016/j.mcm.2011.02.003

[B33] Hill R, Li A, Grutzendler J (2018) Lifelong cortical myelin plasticity and age-related degeneration in the live mammalian brain. Nat Neurosci 21:683–695. 10.1038/s41593-018-0120-6 29556031 PMC5920745

[B34] Hodgkin AL, Huxley AF (1952) A quantitative description of membrane current and its application to conduction and excitation in nerve. J Physiol 117:500–544. 10.1113/jphysiol.1952.sp004764 12991237 PMC1392413

[B35] Hughes EG, Orthmann-Murphy JL, Langseth AJ, Bergles DE (2018) Myelin remodeling through experience-dependent oligodendrogenesis in the adult somatosensory cortex. Nat Neurosci 21:696–706. Published online March 19, 2018. 10.1038/s41593-018-0121-5 29556025 PMC5920726

[B36] Knowles JK, et al. (2022) Maladaptive myelination promotes generalized epilepsy progression. Nat Neurosci 25:596–606. 10.1038/s41593-022-01052-2 35501379 PMC9076538

[B37] Koskinen M, et al. (2023) Node of Ranvier remodeling in chronic psychosocial stress and anxiety. Neuropsychopharmacology 48:1532–1540. 10.1038/s41386-023-01568-6 36949148 PMC10425340

[B38] Krasnow AM, Ford MC, Valdivia LE, Wilson SW, Attwell D (2018) Regulation of developing myelin sheath elongation by oligodendrocyte calcium transients in vivo. Nat Neurosci 21:24–28. 10.1038/s41593-017-0031-y 29230052 PMC6478117

[B39] Lie IA, Weeda MM, Mattiesing RM, Mol MA, Pouwels PJ, Barkhof F, Torkildsen Ø., Bø L, Myhr KM, Vrenken H (2022) Relationship between white matter lesions and gray matter atrophy in multiple sclerosis. Neurology 98:e1562–e1573. 10.1212/WNL.0000000000200006 35173016 PMC9038199

[B40] Mabbott DJ, Noseworthy M, Bouffet E, Laughlin S, Rockel C (2006) White matter growth as a mechanism of cognitive development in children. NeuroImage 33:936–946. 10.1016/j.neuroimage.2006.07.024 16978884

[B41] McIntyre CC, Richardson AG, Grill WM (2002) Modeling the excitability of mammalian nerve fibers: influence of afterpotentials on the recovery cycle. J Neurophysiol 87:995–1006. 10.1152/jn.00353.2001 11826063

[B42] McKenzie I, Ohayon D, Li H, de Faria J, Emery B, Tohyama K, Richardson W (2014) Motor skill learning requires active central myelination. Science 346:318–322. 10.1126/science.1254960 25324381 PMC6324726

[B43] Micheva KD, Wolman D, Mensh BD, Pax E, Buchanan J, Smith SJ, Bock DD (2016) A large fraction of neocortical myelin ensheathes axons of local inhibitory neurons. eLife 5:e15784. 10.7554/eLife.15784 27383052 PMC4972537

[B44] Mount CW, Monje M (2017) Wrapped to adapt: experience-dependent myelination. Neuron 95:743–756. 10.1016/j.neuron.2017.07.009 28817797 PMC5667660

[B45] Nagy N, Westerberg H, Klingberg T (2004) Maturation of white matter is associated with the development of cognitive functions during childhood. J Cogn Neurosci 16:1227–1233. 10.1162/0898929041920441 15453975

[B46] Naud R, Longtin A (2019) Linking demyelination to compound action potential dispersion with a spike-diffuse-spike approach. J Math Neurosci 9:3. 10.1186/s13408-019-0071-6 31147800 PMC6542900

[B47] Nygren A, Halter J (1999) A general approach to modeling conduction and concentration dynamics in excitable cells of concentric cylindrical geometry. J Theor Biol 199:329–358. 10.1006/jtbi.1999.0962 10433897

[B48] Orthmann-Murphy J, Call CL, Molina-Castro GC, Hsieh YC, Rasband MN, Calabresi PA, Bergles DE (2020) Remyelination alters the pattern of myelin in the cerebral cortex. eLife 9:e56621. Published online May 27, 2020; Version of Record: June 15, 2020 10.7554/eLife.56621 32459173 PMC7292648

[B49] Osso LA, Hughes EG (2024) Dynamics of mature myelin. Nat Neurosci 27:1449–1461. Epub May 21, 2024, Review Article 10.1038/s41593-024-01642-2 38773349 PMC11515933

[B50] Pan S, Mayoral S, Choi H, Chan J, Kheirbek M (2020) Preservation of a remote fear memory requires new myelin formation. Nat Neurosci 23:487–499. 10.1038/s41593-019-0582-1 32042175 PMC7213814

[B51] Prins M, Schul E, Geurts J, van der Valk P, Drukarch B, van Dam AM (2015) Pathological differences between white and grey matter multiple sclerosis lesions. Ann N Y Acad Sci 1351:99–113. 10.1111/nyas.12841 26200258

[B52] Pujol J, Soriano-Mas C, Ortiz H, Sebastián-Gallés N, Losilla JM, Deus J (2006) Myelination of language-related areas in the developing brain. Neurology 66:339–343. 10.1212/01.wnl.0000201049.66073.8d 16476931

[B53] Rall W (1969) Time constants and electrotonic length of membrane cylinders and neurons. Biophys J 9:1483–1508. 10.1016/S0006-3495(69)86467-2 5352228 PMC1367649

[B54] Rosenbluth J (2009) Multiple functions of the paranodal junction of myelinated nerve fibers. J Neurosci Res 87:3250–3258. 10.1002/jnr.22013 19224642

[B55] Salami M, Itami C, Tsumoto T, Kimura F (2003) Change of conduction velocity by regional myelination yields constant latency irrespective of distance between thalamus and cortex. Proc Natl Acad Sci 100:6174–6179. 10.1073/pnas.0937380100 12719546 PMC156345

[B56] Schauf CL, Davis FA (1974) Impulse conduction in multiple sclerosis: a theoretical basis for modification by temperature and pharmacological agents. J Neurol Neurosurg Psychiatry 37:152–161. 10.1136/jnnp.37.2.152 4362242 PMC494594

[B57] Schmidt H, Knösche T (2019) Action potential propagation and synchronisation in myelinated axons. PLoS Comput Biol 15:e1007004. 10.1371/journal.pcbi.1007004 31622338 PMC6818808

[B58] Scurfield A, Latimer D (2018) A computational study of the impact of inhomogeneous internodal lengths on conduction velocity in myelinated neurons. PLoS ONE 13:e0191106. 10.1371/journal.pone.0191106 29329312 PMC5766232

[B59] Simons M, Nave K (2015) Oligodendrocytes: myelination and axonal support. Cold Spring Harb Perspect Biol 22:a020479. 10.1101/cshperspect.a020479 26101081 PMC4691794

[B60] Steadman PE, Xia F, Ahmed M, Mocle AJ, Penning AR, Geraghty AC, Steenland HW, Monje M, Josselyn SA, Frankland PW (2020) Disruption of oligodendrogenesis impairs memory consolidation in adult mice. Neuron 105:150–164.e6. 10.1016/j.neuron.2019.10.013 31753579 PMC7579726

[B61] Stedehouder J, et al. (2019) Local axonal morphology guides the topography of interneuron myelination in mouse and human neocortex. eLife 8:e48615. 10.7554/eLife.48615 31742557 PMC6927753

[B62] Sturrock RR (1980) Myelination of the mouse corpus callosum. Neuropathol Appl Neurobiol 6:415–420. 10.1111/j.1365-2990.1980.tb00219.x 7453945

[B63] Takahashi N, Sakurai T, Davis KL, Buxbaum JD (2011) Linking oligodendrocyte and myelin dysfunction to neurocircuitry abnormalities in schizophrenia. Prog Neurobiol 93:13–24. 10.1016/j.pneurobio.2010.09.004 20950668 PMC3622281

[B64] Talidou A, Burchard A, Sigal IM (2021) Near-pulse solutions of the FitzHugh–Nagumo equations on cylindrical surfaces. J NonLinear Sci 31:57. 10.1007/s00332-021-09710-8

[B65] Talidou A, Frankland P, Mabbott D, Lefebvre J (2022) Homeostatic coordination and up-regulation of neural activity by activity-dependent myelination. Nat Comput Sci 2:665–676. 10.1038/s43588-022-00315-z 38177260

[B66] Thornton MA, et al. (2024) Long-term in vivo three-photon imaging reveals region-specific differences in healthy and regenerative oligodendrogenesis. Nat Neurosci 27:846–861. 10.1038/s41593-024-01613-7 38539013 PMC11104262

[B67] Timmler S, Simons M (2019) Grey matter myelination. Glia 67:2063–2070. Epub March 12, 2019, Review Article 10.1002/glia.23614 30860619

[B68] Tomassy GS, Berger DR, Chen HH, Kasthuri N, Hayworth KJ, Vercelli A, Seung HS, Lichtman JW, Arlotta P (2014) Distinct profiles of myelin distribution along single axons of pyramidal neurons in the neocortex. Science 344:319–324. 10.1126/science.1249766 24744380 PMC4122120

[B69] Wang XJ, Buzsáki G (1996) Gamma oscillation by synaptic inhibition in a hippocampal interneuronal network model. J Neurosci 16:6402–6413. 10.1523/JNEUROSCI.16-20-06402.1996 8815919 PMC6578902

[B70] Xin W, Chan J (2020) Myelin plasticity: sculpting circuits in learning and memory. Nat Rev Neurosci 21:682–694. 10.1038/s41583-020-00379-8 33046886 PMC8018611

